# A novel enzymatic method for the measurement of lactose in lactose‐free products

**DOI:** 10.1002/jsfa.9317

**Published:** 2018-10-11

**Authors:** David Mangan, Barry V McCleary, Helena Culleton, Claudio Cornaggia, Ruth Ivory, Vincent A McKie, Elaine Delaney, Tadas Kargelis

**Affiliations:** ^1^ Megazyme u.c., IDA Business Park Southern Cross Road, Bray, Co. Wicklow Ireland

**Keywords:** Lactose determination, Lactose‐free, Enzymatic assay, β‐Galactosidase

## Abstract

**BACKGROUND:**

In recent years there has been a surge in the number of commercially available lactose‐free variants of a wide variety of products. This presents an analytical challenge for the measurement of the residual lactose content in the presence of high levels of mono‐, di‐, and oligosaccharides.

**RESULTS:**

In the current work, we describe the development of a novel enzymatic low‐lactose determination method termed LOLAC (low lactose), which is based on an optimized glucose removal pre‐treatment step followed by a sequential enzymatic assay that measures residual glucose and lactose in a single cuvette. Sensitivity was improved over existing enzymatic lactose assays through the extension of the typical glucose detection biochemical pathway to amplify the signal response. Selectivity for lactose in the presence of structurally similar oligosaccharides was provided by using a *β*‐galactosidase with much improved selectivity over the analytical industry standards from *Aspergillus oryzae* and *Escherichia coli* (EcLacZ), coupled with a ‘creep’ calculation adjustment to account for any overestimation. The resulting enzymatic method was fully characterized in terms of its linear range (2.3–113 mg per 100 g), limit of detection (LOD) (0.13 mg per 100 g), limit of quantification (LOQ) (0.44 mg per 100 g) and reproducibility (≤ 3.2% coefficient of variation (CV)). A range of commercially available lactose‐free samples were analyzed with spiking experiments and excellent recoveries were obtained. Lactose quantitation in lactose‐free infant formula, a particularly challenging matrix, was carried out using the LOLAC method and the results compared favorably with those obtained from a United Kingdom Accreditation Service (UKAS) accredited laboratory employing quantitative high performance anion exchange chromatography – pulsed amperometric detection (HPAEC‐PAD) analysis.

**CONCLUSION:**

The LOLAC assay is the first reported enzymatic method that accurately quantitates lactose in lactose‐free samples. © 2018 The Authors. *Journal of The Science of Food and Agriculture* published by John Wiley & Sons Ltd on behalf of Society of Chemical Industry.

## INTRODUCTION

Lactose is by far the most abundant sugar found in bovine milk, usually present at ∼50 g L^−1^. As a result, it is also typically found in a wide range of commercial dairy products including cheese, yoghurt, cream, ice‐cream, butter, and whey. Following ingestion, lactose is normally hydrolyzed by lactase‐phlorizin hydrolase (commonly referred to as lactase, *β*‐galactosidase, EC. 3.2.1.108) in the human small intestine, with absorption of the released d‐galactose and d‐glucose.^1^ Humans lacking or deficient in this enzyme cannot digest lactose, which therefore passes undigested to the colon where it undergoes microbial fermentation causing adverse gastrointestinal symptoms associated with lactose intolerance, such as nausea, cramps, bloating, and diarrhea.^2^

Lactose intolerance or lactose non‐persistence affects approximately 65% of the global human population. The frequency of primary lactose intolerance varies greatly between ethnic and racial populations, with approximately 5% of northern European and more than 90% of southeast Asian populations being affected. 3, 4

To address the prevalence of lactose intolerance, dairy manufacturers have introduced low‐lactose and lactose‐free dairy products, which are typically manufactured by the addition of a commercial *β*‐galactosidase (EC 3.2.1.23) to hydrolyze the lactose present into glucose and galactose. While the Food and Drug Administration (FDA) and the European Food Safety Authority (EFSA) have not issued overarching regulations on the allowable lactose thresholds in all products, a number of countries have defined ‘low‐lactose’ as containing less than 1 g lactose per 100 g product and ‘lactose‐free’ as containing less than 10–100 mg lactose per 100 g product. In EU legislation, the term ‘lactose‐free’ has been defined only for infant and follow‐on formulas as < 10 mg lactose per 100 kcal.^5^

It is well known that, along with its primary hydrolytic function, *β*‐galactosidase also catalyzes a transglycosylation process in which the released galactose can be transferred to lactose or pre‐formed glucose, galactose, or galacto‐oligosaccharides (GOS). This reaction is employed industrially utilizing high concentrations of lactose to produce GOS as a prebiotic ingredient.^6^, ^7^, ^8^ However, transglycosylation also occurs to some extent during the hydrolysis of lactose for the production of low‐lactose and lactose‐free products. In this process, trace quantities of a range of galactosyl‐glucosyl oligosaccharides are formed. The concentrations of the various transglycosylation products are very low but they can occur at levels similar to, or greater than, that of the residual lactose that is present, which complicates the measurement of lactose in these samples.

The measurement of lactose has been described using a range of methods including IR spectroscopy^9^ (AOAC method 972.16), polarimetry^10^ (AOAC method 896.01), gravimetry^11^ (AOAC method 930.28) along with various types of chromatographic and enzymatic methods. Of these, only chromatographic and enzymatic methods can exhibit the selectivity and/or sensitivity required for the accurate measurement of lactose in low‐lactose or lactose‐free products and hence warrant further discussion.

In employing chromatographic methods, the refractive index^12^ (RI) and evaporative light scattering detectors^13^ (ELSD) lack the sensitivity to quantitate accurately the extremely low level of residual lactose that can be present in a lactose‐free dairy product. Furthermore, the resolution required to separate lactose fully from its associated transglycosylation products cannot be obtained with the commonly employed reverse‐phase or hydrophilic interaction (HILIC) chromatographic methodology.^14^ High‐performance anion exchange chromatography coupled with pulsed amperometric detection (HPAEC‐PAD), on the contrary, does exhibit the required sensitivity and resolution.^15^, ^16^ However, this technique is poorly suited to high‐throughput quality‐control applications in industry due to the prohibitive equipment cost, the high level of expertise needed to operate it correctly and the long duration of the run‐times that it necessitates per sample given that parallel ‘spiked’ samples are required for quantitation.

In the case of enzymatic methods, it is worth noting that a number of groups have experimented with the application of cellobiose dehydrogenase (CDH) to oxidize and measure lactose.^17^, ^18^ This technology has recently been developed into a commercial biosensor but this will not be discussed in detail here, beyond stating its major advantages, namely convenience, sensitivity, and selectivity, and its major disadvantage: the high cost per test (approximately five times that of a standard enzymatic assay) arising from the requirement to use disposable test strips in addition to the initial cost required to purchase the biosensor instrument.

Traditional enzymatic methods have relied on the *β*‐galactosidase‐mediated hydrolysis of lactose and the subsequent measurement of the glucose (or galactose) released as a result.^19^, ^20^ These methods are rapid, easy to perform, and highly cost effective. However, although they are well suited to the measurement of lactose in traditional dairy products in which lactose contents of ∼50 g L^−1^ are usually found, their application to the measurement of lactose in lactose‐free products typically results in inaccurate quantitations with overestimation of the residual lactose present being the most prevalent outcome. These challenges and the solutions developed to overcome them are described in the current work.

## EXPERIMENTAL

### Chemicals and reagents

Glucose (G8270), lactose (17814), hydrogen peroxide (H1009), Carrez I solution (potassium hexacyanoferrate (II) trihydrate (60279)), Carrez II solution (zinc sulfate heptahydrate (31665)), magnesium chloride (M8266), triethylamine (TEA) hydrochloride (T1502), sodium citrate monobasic (71498), citric acid (27488), sodium phosphate monobasic (71496), sodium phosphate dibasic dihydrate (71645), potassium phosphate monobasic (795488), potassium phosphate dibasic (795496), iodonitrotetrazolium chloride (INT) (I8377), flavin adenine dinucleotide (F6625), Triton X‐100 (T9284), and imidazole (I202) were purchased from Sigma‐Aldrich, Arklow, Ireland. Vivinal GOS (Product # 598227; Lot # 704075) was kindly provided by Friesland Campina, Amersfoort, The Netherlands. Allolactose (O‐LAC6), 4‐nitrophenyl‐*β*‐d‐galactopyranoside (O‐PNPBGAL), glucose oxidase/catalase mixture (E‐GOXCA), hexokinase/glucose‐6‐phosphate dehydrogenase mixture (E‐HKGDH), 6‐phosphogluconate dehydrogenase (E‐PGDHEC), diaphorase (E‐DIAEC), EcLacZ (E‐ECBGAL), A. oryzae (E‐BGLAN) and MZ104 *β*‐galactosidase enzyme mixtures were obtained from Megazyme, Bray, Ireland. Note that following the current study, all components required to perform the low‐lactose (LOLAC) assay were made available as an assay kit from Megazyme (K‐LOLAC).

### Enzyme unit definitions

One unit of *β*‐galactosidase activity is defined as the amount of enzyme required to release one µmole of *p*‐nitrophenol (pNP) per minute from *p*‐nitrophenyl‐*β* ‐d‐galactopyranoside (10 mmol L^−1^) in imidazole buffer (100 mmol L^−1^) at pH 7.6 and 40 °C. One unit of catalase activity is defined as the amount of enzyme required to decompose one µmole of H_2_O_2_ per minute while the H_2_O_2_ concentration falls from 10.3 to 9.2 mmol L^−1^, at pH 7.0 and 25 °C. One unit of glucose oxidase activity is defined as the amount of enzyme required to oxidize one µmole of *β* ‐d‐glucose to d‐gluconolactone and H_2_O_2_ per minute, at pH 7.0 at 37 °C. One unit of glucose‐6‐phosphate dehydrogenase activity is defined as the amount of enzyme required to convert one µmole of glucose 6‐phosphate (3.3 mmol L^−1^) to 6‐phosphogluconate per minute in the presence of NAD^+^ in a Tris‐HCl buffer (51 mmol L^−1^), at pH 7.8 and 30 °C. One unit of 6‐phosphogluconate dehydrogenase activity is the amount of enzyme required to convert one µmole of 6‐phosphogluconate to d‐ribulose‐5‐phosphate per minute in TEA buffer (86 mmol L^−1^), pH 7.6 at 25 °C. One unit of hexokinase activity is defined as the amount of enzyme required to produce one µmole of NADH from NAD^+^ in the presence of d‐glucose and glucose‐6‐phosphate dehydrogenase at pH 7.4 and 25 °C. One unit of diaphorase activity is equivalent to a decrease in absorbance of 1.0 per minute at 600 nm with 2,6‐dischlorophenolindophenol and NADH, at pH 7.5 and 25 °C.

### HPAEC‐PAD analysis

Dairy samples were extracted following the Carrez treatment/extraction procedure described below. Galacto‐oligosaccharide samples were analyzed by dilution in Milli‐Q water. The HPAEC‐PAD analysis was performed as described by van Leeuwen *et al*.^7^ using a Dionex ICS‐5000 workstation (Dionex, Amsterdam, Netherlands), equipped with a CarboPac PA‐1 column (250 × 4 mm) with accompanying guard column (50 × 4 mm) and an ICS‐5000 ED pulsed amperometric detector (PAD) at 20 °C using a complex gradient of A: 100 mmol L^−1^ NaOH, B: 600 mmol L^−1^ NaOAc in 100 mmol L^−1^ NaOH, C: Milli‐Q water, and D: 50 mmol L^−1^ NaOAc. The analytical separations were performed at 1.0 mL min^−1^ with 10% A, 85% C, and 5% D in 25 min to 40% A, 10%C, and 50% D, followed by a 35 min gradient to 75% A, 25% B, directly followed by 5 min washing with 100% B and reconditioning for 7 min with 10% A, 85% B, and 5% D.

Note that the analysis of the lactose‐free infant formulas was outsourced to Eurofins ILS, Shardlow Business Park, London Rd, Shardlow, Derby DE72 2GD, UK as the authors are not certified to run UKAS method 30.137.

### Optimization of glucose removal pre‐treatment

Sample clarification was performed using an adapted Carrez treatment protocol taken from the Lactulose Assay Kit (K‐LACTUL) available from Megazyme.

Pre‐treatment optimization to remove excess levels of glucose in samples was performed in both glass test tubes and sealed screw‐capped 13 mL polypropylene tubes (Sarstedt cat. no. 60.541.685). Reactions were carried out in 1.8 mL reaction volumes containing 25–80 mg glucose in 0.1–2.0 mol L^−1^ buffer (triethylamine, citrate, citrate‐phosphate, potassium phosphate, imidazole, tris), 0–20 mmol L^−1^ MgCl_2_, 100–500 U mL^−1^ glucose oxidase (GOX), 500–16 000 U mL^−1^ catalase and 10–40 mg mL^−1^ H_2_O_2_. The incubations were performed at temperatures ranging from 25–40 °C for a set time (5–30 min) and reactions were terminated by incubation at 100 °C for 5 min. Concentration of glucose remaining (g L^−1^) was determined using the standard K‐LOLAC detection method as described below.

### Standard K‐LOLAC assay

#### *Sample preparation/extraction (Carrez treatment)*

Liquid samples: distilled water (0.9 mL), liquid sample (0.5 mL), Carrez solution I (potassium ferrocyanide in water, 0.36 mol L^−1^, 0.05 mL), and Carrez solution II (zinc sulfate in water, 1.04 mol L^−1^, 0.05 mL) were added to a 1.5 mL polypropylene microfuge tube. The tube was capped, mixed by vortexing, and then centrifuged at ∼11 000×*g* for 10 min. The supernatant obtained was used in subsequent steps.

Solid samples: an homogenized sample (10 g) was added to a 50 mL glass beaker followed by distilled water (approximately 30 mL). The mixture was heated to approximately 50 °C and stirred using a magnetic stirrer for 15 min. Carrez solution I (potassium ferrocyanide in water, 0.36 mol L^−1^, 0.5 mL) and Carrez solution II (zinc sulfate in water, 1.04 mol L^−1^, 0.5 mL) were added to this and the mixture was then quantitatively transferred to a 50 mL volumetric flask where it was diluted to volume (50 mL) with distilled water. Mixtures were then stirred thoroughly and filtered through Whatman No. 1 filter paper. The clear solution obtained was used in subsequent steps.

#### *Glucose removal pre‐treatment*

Distilled water (0.40 mL), supernatant obtained from sample preparation/extraction (1.00 mL), imidazole buffer (5 mol L^−1^, pH 8, 0.10 mL) containing 5 g L^−1^ MgCl_2_, glucose oxidase (400 U) / catalase (3400 U) solution (0.20 mL) and hydrogen peroxide (10 mol L^−1^, 0.10 mL) were added to a 13 mL polypropylene tube. The tube was immediately capped and the contents were mixed well. After 15 min, the pressure was released by carefully loosening the cap before re‐tightening and incubating at 100 °C for 5 min. The solution was transferred to a 1.5 mL microfuge tube and centrifuged at 11 000×*g* for 10 min. The supernatant obtained was used in subsequent steps.

#### *Glucose/lactose enzymatic determination*

Enzymatic d‐glucose/lactose determination assays were performed in 1.5 mL semi‐micro cuvettes. Final reaction volume was 1.15 mL containing 0.5–25 µg of d‐glucose or 1–50 µg of lactose, 150 mmol L^−1^ imidazole (pH 7.6), 8 mmol L^−1^ MgCl_2_, 0.9 g L^−1^ NADP^+^ and 1.8 g L^−1^ ATP. Initial absorbances (A1) were measured spectrophotometrically at 340 nm. Measurement of residual d‐glucose was initiated with the addition of 20 µL enzyme mixture containing hexokinase (425 U mL^−1^), glucose‐6‐phosphate dehydrogenase (150 U mL^−1^) and 6‐phosphogluconate dehydrogenase (50 U mL^−1^). Absorbances (A2) were measured spectrophotometrically at 340 nm after 10 min incubation (or when reaction had reached completion). The lactose determination reaction was then initiated with the addition of 20 µL of MZ104 *β*‐galactosidase (3500 U mL^−1^). Absorbances (A3) were measured spectrophotometrically at 340 nm after 15 min incubation (or when reaction had reached completion). In those instances where the reaction did not reach completion after 15 min, absorbances were read at 5 min intervals until the absorbances either remained unchanged (reaction completion) or continued to increase in a linear fashion with respect to time (creep). If this ‘creep’ rate was greater for the sample than for the blank, sample absorbance was extrapolated back to the time of the addition of *β*‐galactosidase. This can be simplified using the Megazyme MegaCalc tool for creep calculation (www.megazyme.com). *Δ*A_D‐glucose_ was obtained by subtracting (A2 − A1_blank_) from (A2 − A1_sample_). *Δ*A_lactose_ was obtained by subtracting (A3 − A2_blank_) from (A3 − A2_sample_). Both d‐glucose and lactose concentrations are expressed in g L^−1^ or g per 100 g and can be calculated as follows:

$$g\;L^{-1}=\frac{V*\mathrm{MW}}{\epsilon *d*v*2}*\;F\;*\;\Delta A$$

$$g\;\mathrm{per}\;100\;g=\frac{\;\text{lactose concentration}\;\left(g\;L^{-1}\right)\;\;}{\text{sample weight}\;\left(g\right)}\;*\;100$$

where:

V = final volume in cuvette [mL], MW = molecular weight of d‐glucose or lactose [g mol^−1^], ϵ = extinction coefficient of NADPH at 340 nm = 6300 mol^−1^ cm^−1^, d = light path [cm], v = sample volume in cuvette [mL], 2 = 2 moles of NADPH produced for each mole of d‐glucose or lactose, F = dilution factor (see Fig. S1 in the supporting information), *Δ*A = (A3 − A2_sample_) – (A3 − A2_blank_)

### Optimization of EcLacZ and MZ104 *β*‐galactosidase content required for quantitative hydrolysis of lactose standard

The standard LOLAC assay procedure was followed as described in the previous section using 0.2 mL of a lactose standard solution (0.25 g L^−1^) as a sample while varying the EcLacZ content from 0.47–9.5 U assay^−1^ (Fig. 4(a)) and the MZ104 content from 2.75 – 44 U assay^−1^ (Fig. 4(b)), while maintaining 20 µL *β*‐galactosidase in the assay. Reactions were monitored over 30 min. 7 U assay^−1^ (20 µL, 350 U mL^−1^) EcLacZ and 33 U assay^−1^ (20 µL, 1650 U mL^−1^) MZ104 were shown to quantitatively hydrolyze 50 µg of lactose.

### Investigation into the selectivity of EcLacZ and MZ104 *β*‐galactosidase on a range of *β*‐galactosidase substrates

Selectivity investigations were performed using the standard K‐LOLAC enzymatic assay described above, using both MZ104 and EcLacZ *β*‐galactosidases. Biogel P2 chromatography was used to separate Vivinal GOS Powder into DP fractions (2‐4) as described previously^6^ and solutions of each were prepared at a concentration of 0.50 g L^−1^ where expected glucose recoveries were as follows: DP2 (0.25 g L^−1^), DP3 (0.167 g L^−1^) and DP4 (0.125 g L^−1^). Lactose and allolactose standard solutions were prepared at a concentration of 0.50 g L^−1^ (expected glucose recovery 0.25 g L^−1^).

### INT/diaphorase high‐sensitivity detection method

Enzymatic d‐glucose/lactose determination assays were performed in 1.5 mL semi‐micro cuvettes. Final reaction volume was 1.21 mL containing 0 and 10 µg of lactose, 100 mmol L^−1^ glycylglycine buffer (pH 8.0), 10 mmol L^−1^ MgCl_2_, 10 g L^−1^ Triton X‐100, 0.1 g L^−1^ flavin adenine dinucleotide, 0.4 g L^−1^ iodonitrotetrazolium chloride, 0.9 g L^−1^ NADP^+^ and 1.8 g L^−1^ ATP. Initial absorbances (A1) were measured spectrophotometrically at 492 nm. d‐Glucose determination reaction was initiated with the addition of 20 µL enzyme mixture containing hexokinase (425 U mL^−1^), glucose‐6‐phosphate dehydrogenase (212 U mL^−1^), 6‐phosphogluconate dehydrogenase (50 U mL^−1^) and diaphorase (200 U mL^−1^). Absorbances (A2) were measured spectrophotometrically at 492 nm after 10 min incubation (or when reaction had reached completion). Lactose determination reaction was then initiated with the addition of 20 µL of MZ104 *β*‐galactosidase (9000 U mL^−1^). Absorbances (A3) were measured spectrophotometrically at 492 nm after 15 min incubation.

## RESULTS AND DISCUSSION

### Removal of residual glucose or galactose

Given that dairy products typically contain ∼50 g L^−1^ lactose it follows that lactose‐free products that have been manufactured through a *β*‐galactosidase treatment contain ∼25 g L^−1^ each of glucose and galactose and usually <1 g L^−1^ lactose. Measurement of the glucose or galactose released from the residual lactose hydrolysis during the assay procedure is challenging for the simple reason that a minuscule increase in the monosaccharide content would be difficult to accurately quantitate relative to the large ‘background’ signal arising from the glucose or galactose already present. Effective removal of this background signal was identified as a key requirement.

**Figure 1:**
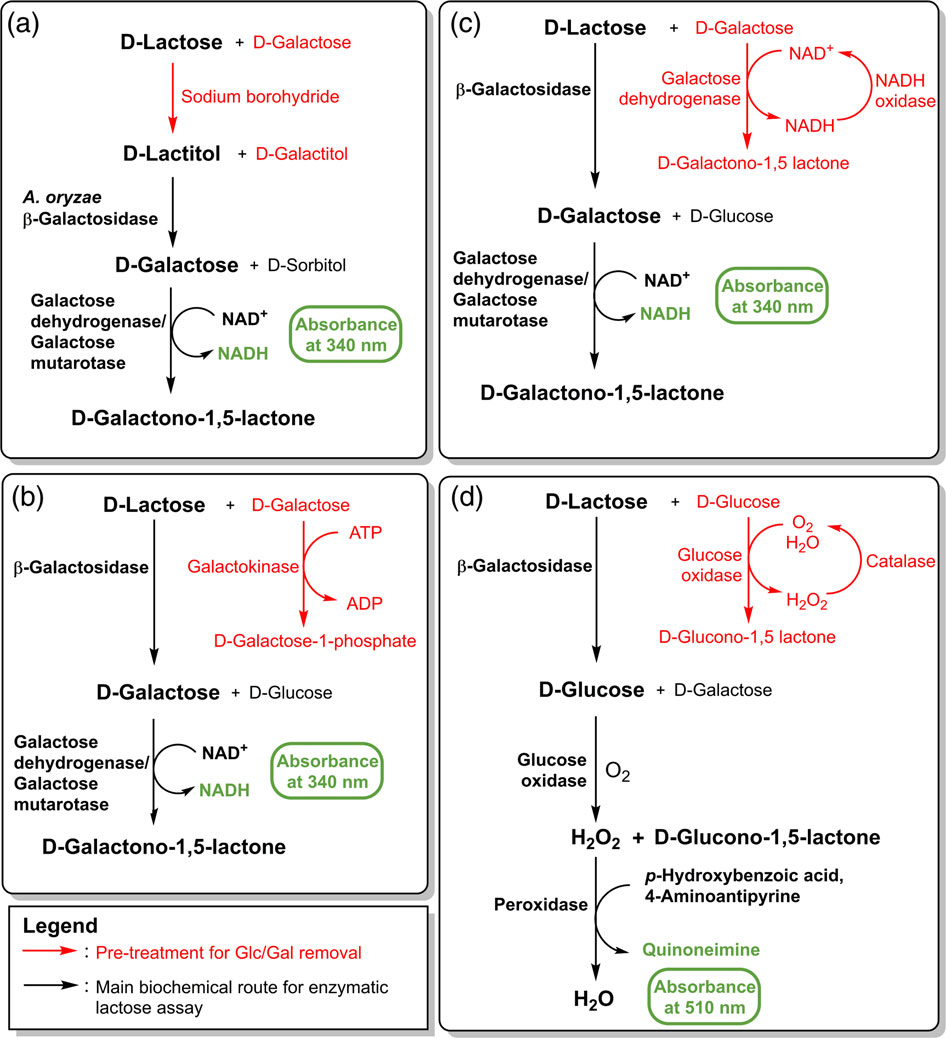
Alternative pre‐treatment steps to remove d‐galactose and d‐glucose that were investigated as part of the current study.

In the case of galactose determination, an assay procedure developed by McCleary *et al*.^21^ which involves pre‐treatment using borohydride reduction of a sample which quantitatively reduced lactose to lactitol, glucose to sorbitol, and galactose to galactitol was previously described. Subsequent hydrolysis of lactitol using a *β*‐galactosidase from *A. oryzae* released galactose that could be measured accurately via a galactose dehydrogenase (EC 1.1.1.48)/galactose mutarotase (EC 5.1.3.3) detection system in the absence of a background signal (Fig. 1(a)).^21^ For reasons that will be discussed in detail later, this broad spectrum *β*‐galactosidase did not exhibit a suitable selectivity profile for the assay of lactose in lactose‐free products. Those *β*‐galactosidases with improved selectivity that were identified as potential replacement candidates were found to be relatively inactive on lactitol, which precluded the use of the borohydride reduction pre‐treatment. Two alternative strategies were investigated to remove galactose prior to analysis. These included the galactokinase mediated approach to convert galactose to galactose‐1‐phosphate as outlined in Fig. 1(b) and the galactose dehydrogenase/NADH oxidase (EC 1.6.3.4) coupled approach to convert galactose to galactono‐1,5‐lactone as shown in Fig. 1(c). Despite extensive optimization (which will not be discussed here), it became clear that neither enzymatic system could achieve the desired outcome in a cost‐effective manner within an acceptable timeframe. Despite initial results that were encouraging, both processes were ultimately challenged by the stoichiometric nature of the biochemistry involved necessitating the use of prohibitively large quantities of ATP and NAD^+^ respectively.

In the case of lactose measurement via glucose determination, a well known catalytic system involving glucose oxidase (EC 1.1.3.4)/catalase (EC 1.11.1.6) (Fig. 1(d)) was investigated. This biochemistry has been employed in the food industry for many years to remove oxygen from food packaging or glucose from egg white to prevent browning.^22^ A ‘glucose remover’ glucose oxidase/catalase reagent was commercially available but manufacturer guidelines recommended a 3 h incubation time, which was deemed unacceptable. A series of optimization experiments were carried out as described above. The importance of maintaining a steady, low concentration of oxygen in the system to facilitate the catalytic cycle was immediately apparent. This necessitated the use of an unexpectedly low initial concentration of catalase (1890 U mL^−1^) along with sealed, screw‐cap vials to prevent an initial rapid release of oxygen upon addition of H_2_O_2_ before the vial could be tightly closed. Given that gluconic acid was produced in a 1:1 molar ratio with concomitant removal of glucose, a 0.3 mol L^−1^ imidazole buffer was identified as the most suitable buffer to maintain pH close to the optima for glucose oxidase and catalase (∼pH 8). The optimized pre‐treatment system has been demonstrated to remove glucose from samples containing up to 80 g L^−1^ glucose in 15 min – which is more than sufficient for the vast majority of lactose‐free dairy samples. If required, larger quantities of glucose can be fully removed by increasing the pre‐treatment incubation time.

### Detection method

**Figure 2:**
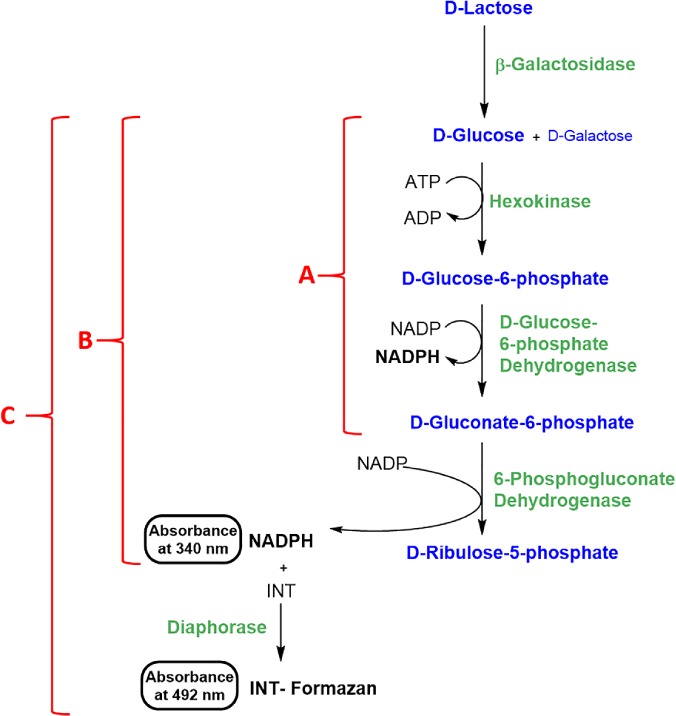
Alternative enzymatic glucose detection methods that were investigated as part of the current study.

Commonly employed detection methods for glucose and galactose are shown in Fig. 1. An alternative, commonly employed method for glucose quantification is outlined in Fig. 2(a), which is based on the use of hexokinase (EC 2.7.1.1) and glucose‐6‐phosphate dehydrogenase (EC 1.1.1.49).^23^ Using this biochemical pathway, 1 mole of glucose generates 1 mole of NADPH, which absorbs strongly at 340 nm. To increase the sensitivity of the current assay and therefore lower the limit of detection (LOD) and limit of quantification (LOQ), an additional enzymatic step employing 6‐phosphogluconate dehydrogenase (EC 1.1.1.44) was added to the biochemical pathway as shown in Fig. 2(b). This modification effectively increases the sensitivity of the assay two‐fold given that 2 moles of NADPH are now generated for each mole of glucose. Even though the sensitivity provided by this modification was thought to be sufficient for the vast majority of lactose assays, a further sensitivity enhancement was also investigated to demonstrate the maximum sensitivity obtainable for a traditional UV spectrophotometer. Figure 2(c) outlines the extension of the existing biochemical pathway using diaphorase (NADPH dehydrogenase; EC 1.6.99.1) to quantitatively oxidize the NADPH formed with the concomitant reduction of 2‐(4‐iodophenyl)‐3‐(4‐nitrophenyl)‐5‐phenyl‐2H‐tetrazolium (INT) to the corresponding formazan dye. The stoichiometry with respect to glucose remains unchanged but a significant gain in observed sensitivity is obtained through the substantial difference in the molar extinction coefficient of INT (19 900 mol L^−1^ cm^−1^ at 492 nm) versus NADPH (6300 mol L^−1^ cm^−1^ at 340 nm) (Fig. S2 in supporting information). The experimental details are discussed above but, following a cost/benefit analysis, the biochemistry outlined in Fig. 2(b) was considered the most suitable detection method for the standard lactose assay procedure.

### *β*‐Galactosidase selectivity

**Figure 3:**
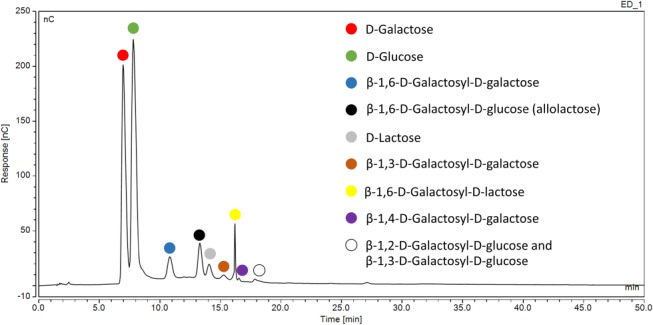
Typical HPAEC‐PAD profile obtained for a lactose‐free dairy product. Note that the peaks corresponding to glucose and galactose are ‘off scale’ and therefore grossly underestimated in this chromatogram. Analysis at this concentration was necessary to achieve a sufficient detector response for residual lactose and GOS.

As discussed in the introduction, measurement of lactose in the presence of its associated transglycosylation products poses a significant challenge for traditional enzymatic methods. Analysis of a sample set of commercially available lactose‐free products by HPAEC‐PAD was performed as described above to determine whether a ‘typical’ oligosaccharide profile could be identified in lactose‐free dairy foods and beverages. The relative concentration of the various transglycosylation species was observed to vary considerably from sample to sample but certain key features were ubiquitous. Allolactose (*β*‐1,6‐d‐glucosyl‐d‐galactose) was consistently present as the most abundant oligosaccharide with *β*‐1,6‐d‐galactosyl‐d‐galactose, *β*‐1,4‐d‐galactosyl‐d‐galactose and *β*‐1,6‐d‐galactosyl‐lactose also normally present in appreciable quantities. *β*‐1,3‐d‐Galactosyl‐d‐galactose, *β*‐1,2‐d‐galactosyl‐d‐glucose and *β*‐1,3‐d‐glucosyl‐d‐glucose were present in a smaller number of samples and usually only in trace quantities. Figure 3 shows a representative example of the chromatographs obtained. It should be apparent that d‐galactosyl residues are present in higher abundance than d‐glucosyl residues across the transglycosylation product profile. This suggested that an assay based on d‐glucose measurement (as opposed to d‐galactose measurement) would result in a reduction of the overestimation of lactose content obtained following incubation with a non‐specific *β*‐galactosidase. When taken in combination with the rapid, efficient, catalytic background glucose‐removal technique described, a clear preference for an assay based on d‐glucose detection materialized.

Theoretically, by using a *β*‐galactosidase with strict specificity for lactose, it should be possible to quantitatively hydrolyze the residual lactose in a sample and not hydrolyze any of the related transglycosylation products. In reality, given that *β*‐galactosidases tend to exhibit a shallow binding pocket capable of binding only a single galactose residue it was thought to be unlikely that such an enzyme existed in nature, although some examples are described with preferential selectivities.^24^, ^25^, ^26^ The two *β*‐galactosidases routinely employed in commercial lactose assay kits are *A. oryzae β*‐galactosidase and *E. coli β*‐galactosidase (EcLacZ). In the current study, their selectivities were compared with that of a novel *β*‐galactosidase not previously reported, hereafter referred to as MZ104. To carry out a selectivity study, a number of *β*‐galactosidase substrates were employed including lactose, allolactose, and a series of GOS mixtures, which were fractionated by DP from commercially available Vivinal GOS using Biogel‐P2 chromatography as described above. From the initial experiments, it was clear that the *A. oryzae β*‐galactosidase was not suitable for use in a selective lactose assay as it exhibited no selectivity across those substrates evaluated, hydrolyzing all DP GOS fractions, allolactose, and lactose to the same extent.

The results of the selectivity comparison between EcLacZ and MZ104 are outlined in Table 1. It is clear from the data that EcLacZ exhibits some level of selectivity, quantitatively hydrolyzing the DP2 GOS fraction, with the DP3 and DP4 GOS fractions hydrolyzed to a degree of 13% and 1% respectively. No preferential selectivity of lactose over allolactose was observed, consistent with previous reports.^27^ Interestingly, while MZ104 did not display the same level of selectivity for DP2 over higher DP GOS fractions as observed for EcLacZ, it did exhibit significant selectivity for lactose over allolactose. The HPAEC‐PAD analysis also confirmed that the DP2 GOS fraction investigated contained ∼160 g kg^−1^ allolactose, which was resistant to hydrolysis by MZ104 *β*‐galactosidase, along with 370 g kg^−1^ lactose, 380 g kg^−1^ 1,2‐lactose with the remainder being composed of minor quantities of 1,4‐galactobiose and 1,3‐lactose (Fig. S3 in supporting information). Given that allolactose is consistently present in relatively high abundance in low‐lactose products, it was thought that this particular selectivity feature could prove extremely useful.

**Table 1:** Investigation into the selectivity observed for EcLacZ and MZ104 *β*‐galactosidases on a range of *β*‐galactosidase substrates

		Glucose released in *β*‐galactosidase incubation (g L^−1^)		Degree of hydrolysis with *β*‐galactosidase (%)	
Substrate (0.5 g L^−1^)	Predicted glucose content (g L^−1^)^a^	EcLacZ	MZ104	EcLacZ	MZ104
Lactose	0.250	0.249	0.253	100	101
GOS DP2	0.250	0.247	**0.213**	99	**85**
GOS DP3	0.167	0.022	0.090	13	54
GOS DP4	0.125	0.001	0.013	1	10
Allolactose	0.250	0.252	**0.061**	101	**24**

a Note that degree of hydrolysis is calculated based on the predicted glucose content after making the assumption that the ‘average’ oligosaccharide present in each fraction consists of a terminal glucose residue with chain extension composed entirely of galactose residues.

**Figure 4:**
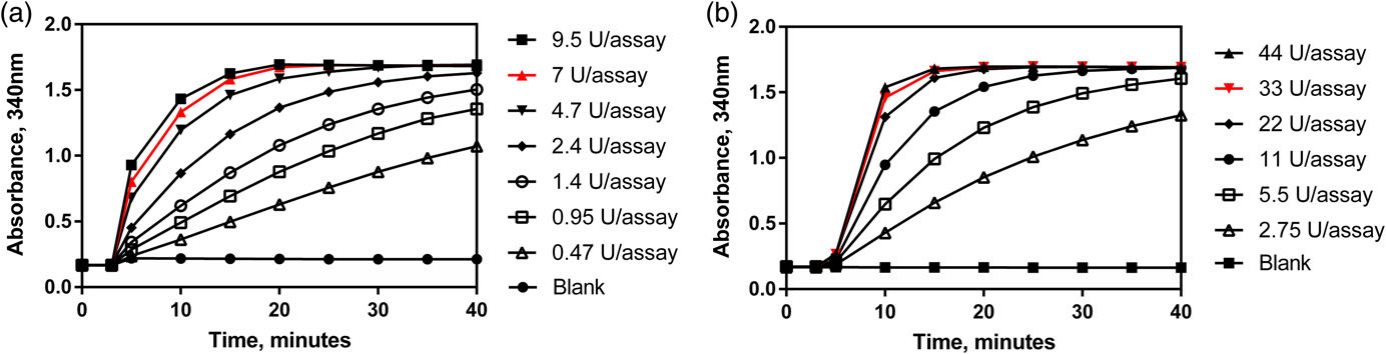
Optimization of (a) EcLacZ and (b) MZ104 *β*‐galactosidase content required for hydrolysis of 50 µg lactose standard under standard LOLAC assay conditions.

**Figure 5:**
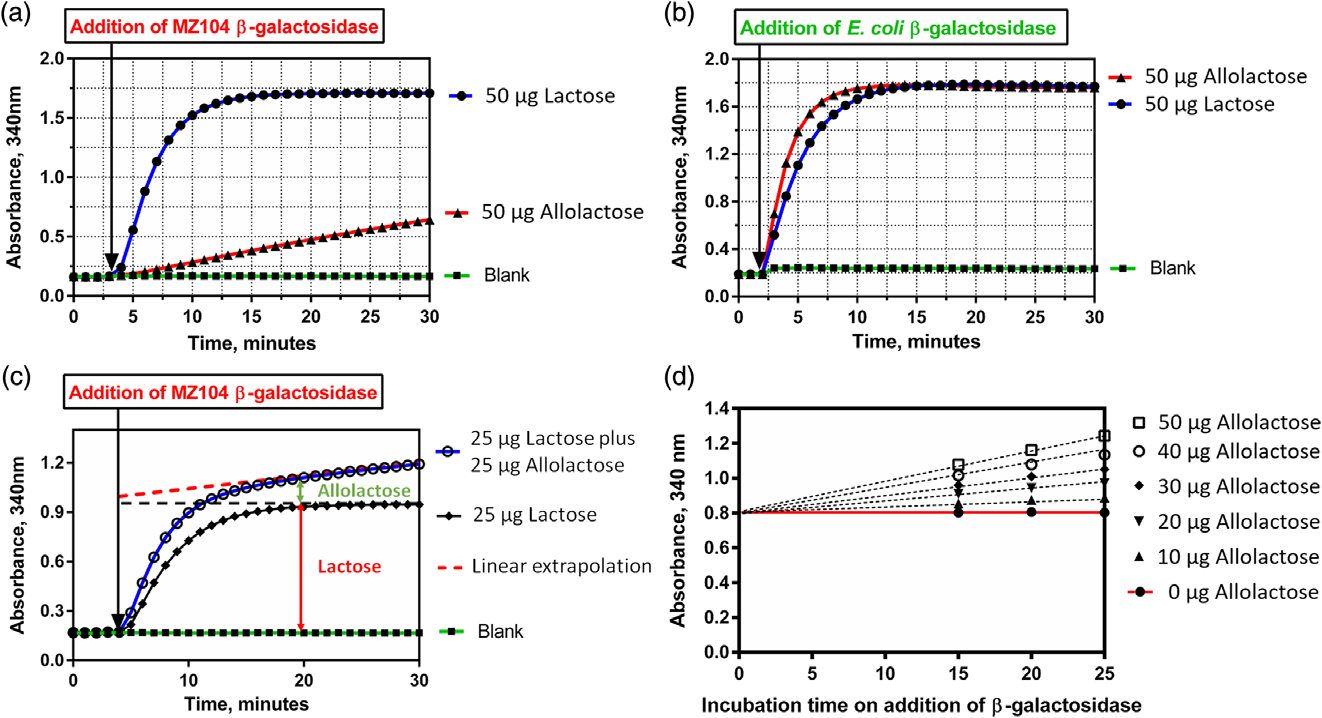
Hydrolysis of 50 µg lactose and allolactose by (a) 33 U MZ104 and (b) 7 U EcLacZ *β*‐galactosidases. (c) LOLAC assay profile for a sample containing 25 µg lactose and 25 µg allolactose with demonstration of the linear extrapolation step to account for overestimation of lactose arising from allolactose hydrolysis. (d) Demonstration of the general application of the creep calculation adjustment for a range of samples containing 25 µg lactose and 0–50 µg allolactose.

Optimization experiments outlined above were used to determine the number of units of MZ104 (33 U assay^−1^) and EcLacZ (7 U assay^−1^) required to quantitatively hydrolyze 50 µg of lactose within 15 min, arbitrary values for lactose quantity and incubation time that were chosen to be the upper limits for the desired assay (Fig. 4(a),(b)). At this point, the decision was taken to deviate from traditional lactose assay formats, which require a separate analysis for glucose/galactose present in a given sample and glucose/galactose released upon hydrolysis of lactose. The new assay described here was designed to operate under a sequential format, meaning that the *β*‐galactosidase is added to the same cuvette assay that was used to determine the background glucose. Following the 15 min *β*‐galactosidase incubation a second absorbance reading can then be taken and the lactose content calculated by subtraction. Under this format, the selectivity difference between MZ104 and EcLacZ for lactose versus allolactose was clearly demonstrated (Fig. 5(a),(b)). The assay profile obtained for a sample containing 25 µg of lactose and 25 µg of allolactose is shown in Fig. 5(c). It was immediately apparent that the linear ‘creep’ reaction observed was due to the continued, slow hydrolysis of allolactose. It was also apparent that linear regression could be employed to extrapolate back to the point of addition of MZ104 *β*‐galactosidase to account for the absorbance contribution of allolactose that would otherwise lead to an overestimation of lactose content (Fig. 5(c)). To demonstrate the broader application of this approach, hydrolysis experiments were conducted across a series of samples in which the lactose content was maintained at 25 µg while the allolactose content was varied from 0 to 50 µg (Fig. 5(d)). By way of extrapolation from the linear region of the reaction profile after the initial 15 min incubation, the overestimation of the lactose present can be completely avoided. The combination of the preferential selectivity of MZ104 for lactose over allolactose coupled with the ‘creep’ adjustment calculation, form the basis for a highly selective assay for lactose in the presence of allolactose.

#### *Method validation*

The key assay features outlined above, namely (i) the optimized glucose removal pre‐treatment; (ii) the ‘double oxidation’ glucose detection system; (iii) the sequential assay format; and (iv) the selectivity increase afforded by the use of MZ104 in conjunction with the creep adjustment, when taken together constitute the basis for the newly developed LOLAC assay.

#### *Linearity and working range*

The ‘in assay’ working range was demonstrated as 0.1–25 µg of glucose (or 0.2–50 µg of lactose), which corresponds to a maximum absorbance change of approximately 1.5 (Fig. S4 in the supporting information). Based on these figures, the standard 0.1 mL sample volume is recommended for low‐lactose products (< 1 g lactose per 100 g) while the standard sample volume can be increased to 0.4 mL (while keeping the total assay volume constant through reduction in the distilled water employed) for lactose‐free products (< 10 mg lactose per 100 g) (Fig. S5 in the supporting information). This then relates back to an effective working range of 14 – 675 g L^−1^ for liquid samples or 2.3–113 mg per 100 g for solid or semi‐solid samples (requiring extraction). These values are derived from the standard sample preparation instruction described above.

#### *LOD and LOQ*

The ‘in assay’ LOD and LOQ for the maximum 0.4 mL sample size were calculated as 0.15 and 0.25 mg L^−1^ respectively, which correspond to 3 × *σ* and 10 × *σ* respectively, where *σ* is the standard deviation observed for 30 replicates of a sample blank. As above, the sample pre‐treatment/extractions steps need to be accounted for in real sample analysis. This leads to an LOD and LOQ of 0.79 and 2.65 mg L^−1^ respectively for liquid samples and 0.13 mg per 100 g and 0.44 mg per 100 g respectively for solid or semi‐solid samples. These values are derived from the standard sample preparation instruction described above.

#### *Reproducibility*

The reproducibility of the LOLAC assay was investigated for standard liquid samples (which includes the recommended pre‐treatment steps) by having two analysts perform duplicate analysis of a range of standard samples on two consecutive days. The range of samples comprised seven solutions with lactose concentrations of 27 mg L^−1^ to 2.7 g L^−1^. The results are outlined in Table 2. Excellent coefficients of variation (CVs) (≤ 3.11%) are obtained down to 54 mg L^−1^ using 0.1 mL sample volume which could be extended to 13.5 mg L^−1^ if 0.4 mL sample volume was employed. The value of 6.22% CV obtained at the 27 mg L^−1^ level is also acceptable given the extremely low signal response obtained.

**Table 2:** Reproducibility (intermediate precision) obtained using a range of lactose standards in the standard liquid sample LOLAC assay procedure

Number of analyses	µg Lactose in cuvette	Ref material (g L^−1^)	Mean value (g L^−1^)	Standard deviation	%CV
8	50	2.7	2.725	0.0357	1.31
8	40	2.16	2.166	0.0232	1.07
8	20	1.08	1.090	0.0093	0.86
8	10	0.54	0.554	0.0103	1.85
8	2	0.108	0.109	0.0030	2.78
8	1	0.054	0.055	0.0017	3.11
8	0.5	0.027	0.026	0.0016	6.22

### Analysis of lactose‐free samples

In order to demonstrate the efficacy of the LOLAC assay method, a range of commercial low‐lactose and lactose‐free products were analyzed using the standard LOLAC assay protocol outlined above. Following pre‐treatment, these samples were also spiked with known quantities of lactose and a comparison of the spiked versus unspiked samples was performed. Failure to quantitatively recover the lactose spikes in a number of samples demonstrated the presence of an inhibitor of MZ104 in the LOLAC assay. Further investigation revealed that a galactose content of > 4 mg assay^−1^ (corresponding to >54 g L^−1^ in the original liquid samples for 0.4 mL sample size) was the source of this inhibition. Although ∼50 g L^−1^ galactose content would certainly be above the level typically found in most commercial samples, it was decided to increase the *β*‐galactosidase activity used in the standard LOLAC assay format from 33 to 70 U assay^−1^ to build in capacity against this potential source of inhibition. The commercial samples were reanalyzed under the updated LOLAC assay format and the results are shown in Table 3. Spiking of liquid samples resulted in excellent recoveries of 96–102% while more challenging matrices such as solid dairy samples resulted in good recoveries of 91–102%. Non‐dairy samples that posed handling issues such as troublesome extraction and/or filtration were also spiked prior to sample pre‐treatment, which resulted in acceptable recoveries of 105–120%.

**Table 3:** Determination of lactose in a range of low‐lactose/lactose‐free products

Sample	Physical form	Lactose in sample (g L^−1^)	Recovery of cuvette spike (%)	Recovery of sample spike (%)
Adult nutritional shake	Liquid	0.83	96.21	—
Kefir	Liquid	40.68	100.4	—
Low‐lactose milk	Liquid	0.58	101.2	—
Low‐lactose milk	Liquid	1.5	99.5	—
Low‐lactose UHT milk	Liquid	0.016	101.4	—
Rice milk	Liquid	0.003	96.5	—
Soya milk	Liquid	0.02	97.4	—
Brie cheese	Solid	0.0010	98.1	—
Cheddar cheese	Solid	0.0031	91.6	—
Coffee creamer	Solid	0.0014	97.9	—
Cottage cheese	Solid	3.22	95.4	—
Natural live yoghurt	Solid	3.22	99.5	—
Parmesan cheese	Solid	0.0004	97.6	—
Processed cheese slices	Solid	4.72	96.6	—
Ricotta cheese	Solid	2.98	96.2	—
Low‐lactose cookies	Solid	0.001	99.5	105.6
Low‐lactose nachos	Solid	0.002	100.4	111.3
Low‐lactose pudding	Solid	0.005	102.00	108.9
Low‐lactose tart	Solid	0.004	100.9	119.9

All assays were spiked with a lactose standard to confirm that full recovery was obtained.

### Lactose‐free infant formula samples

Stringent EU legislation dictates that the upper allowable threshold for lactose in lactose‐free infant formula is 10 mg per 100 kcal.^28^ It is also known that these products often contain relatively high levels of GOS (with respect to lactose) as prebiotics and that these oligosaccharides can be extensively hydrolyzed by the *β*‐galactosidase employed in any enzymatic lactose assay resulting in an overestimation of the lactose present. As such, the determination of lactose in lactose‐free infant formula represents the ‘acid test’ of the LOLAC analytical method. Six representative samples from four European markets were analyzed by three methods, namely (i) using HPAEC‐PAD analysis performed by UKAS method 30.137 at an analytical laboratory in the UK; (ii) the standard LOLAC assay format using MZ104 *β*‐galactosidase; and (iii) the standard LOLAC assay format with MZ104 *β*‐galactosidase replaced by EcLacZ, the *β*‐galactosidase commonly used in commercially available enzymatic assay kits. The results are outlined in Table 4, which also shows the lactose value stated on the manufacturer's nutrition facts label.

**Table 4:** Determination of lactose in lactose‐free baby formula using (a) the standard LOLAC assay format, (b) the standard LOLAC assay format using EcLacZ in place of MZ104 and (c) HPAEC‐PAD analysis using UKAS method 30.137 performed by a UKAS accredited analytical laboratory

Lactose‐free infant formula sample	Product label lactose content (mg per 100 g)	Analysis method	Lactose (mg per 100 g)	Lactose (mg per 100 kcal)	Spike recovery (%)
A	Not stated	MZ104	24.7	4.8	100.2
		EcLacZ	**55.4**	**10.8**	**99.5**
		HPAEC‐PAD	16.5	3.2	108.0
B	50	MZ104	12.5	2.5	99.2
		EcLacZ	46.0	9.0	85.9
		HPAEC‐PAD	9.8	1.9	97.0
C	Not stated	MZ104	19.1	3.8	99.0
		EcLacZ	36.4	7.3	100.5
		HPAEC‐PAD	13.6	2.7	97.0
D	Not stated	MZ104	4.0	0.8	99.0
		EcLacZ	41.7	8.4	106.6
		HPAEC‐PAD	<1	N/A	98.0
E	< 42	MZ104	33.0	7.2	102.2
		EcLacZ	36.0	7.8	90.2
		HPAEC‐PAD	35.3	7.7	106.0
F	< 52	MZ104	6.7	1.3	101.5
		EcLacZ	21.3	4.1	95.5
		HPAEC‐PAD	4.3	0.8	100.0

Minor overestimation of lactose is observed for the LOLAC assay (MZ104 *β*‐galactosidase) when compared to the HPAEC‐PAD method for the majority of these samples. This is almost certainly due to the minor hydrolysis of *β*‐1‐2, *β*‐1‐3 and *β*‐1‐4 linked GOS. The degree of overestimation observed when employing the EcLacZ *β*‐galactosidase is far more pronounced suggesting that *β*‐1,6 linked GOS (including allolactose) constitutes the bulk of the GOS present. It is noteworthy that samples B, C, and D, when analyzed using EcLacZ (mimicking current commercially available enzymatic assays), erroneously report lactose values approaching the 10 mg per 100 kcal threshold whereas sample A actually exceeds this limit and thus would have been incorrectly classified as in breach of the EU regulation. The values observed employing the LOLAC assay are broadly in line with those obtained using the accredited HPAEC‐PAD method.

## CONCLUSION

This article described the development of the first enzymatic assay procedure that accurately measures lactose in lactose‐free products. This was achieved by addressing each of the limitations of existing enzymatic lactose assays, most notably satisfying the key requirements of selectivity and sensitivity. The resulting method was characterized in terms of its working range, linearity, LOD, LOQ, and reproducibility, and the quantitation of lactose in a range of commercially available lactose‐free products was demonstrated with spiking experiments.

The application of the assay to a particularly challenging sample matrix, lactose‐free infant formula, was then investigated and excellent agreement with UKAS method 30.137 (HPAEC‐PAD analysis method) was obtained. While HPAEC‐PAD analysis of lactose in lactose‐free infant formula may offer the most accurate quantitation, the LOLAC assay is far superior to the enzymatic assay kits currently commercially available. Given that HPAEC‐PAD analysis requires significant initial capital investment, highly trained personnel to operate the instrumentation, and typically requires > 2 h per analysis, it could be argued that the LOLAC enzymatic assay, with its simple, robust, rapid format should find much wider application throughout the food and beverage industry.

## Supporting information

Figure S1. Dilution factor calculation for liquid and solid samples based on the recommended procedures.
Figure S2. Absorbance response observed for X µg lactose in the standard LOLAC assay procedure (NADH response) and in the modified LOLAC assay procedure using the glucose detection system described.
Figure S3. HPAEC‐PAD chromatograph obtained for the analysis of the DP2 fraction obtained from Vivinal GOS.
Figure S4. Analysis of the linearity observed for the standard LOLAC assay as described using lactose (0.2–50 µg assay^−1^) and glucose (1–25 µg assay^−1^).
Figure S5. Analysis of the linearity observed for the standard LOLAC assay as described when the sample volume employed for a range of commercial dairy products was varied from 0.1 to 0.4 mL.
