# Supplementary material for: A novel enzymatic method for the measurement of lactose in lactose‐free products
Source: J Sci Food Agric. 2018 Oct 11;99(2):947–56. doi: 10.1002/jsfa.9317 (PMC6585930; doi:10.1002/jsfa.9317)
Supplement: Supplementary file 1 — Figure S1. Dilution factor calculation for liquid and solid samples based on the recommended procedures. Figure S2. Absorbance response observed for X µg lactose in the standard LOLAC assay procedure (NADH response) and in the modified LOLAC assay procedure using the glucose detection system described. Figure S3. HPAEC‐PAD chromatograph obtained for the analysis of the DP2 fraction obtained from Vivinal GOS. Figure S4. Analysis of the linearity observed for the standard LOLAC assay as described using lactose (0.2–50 µg assay−1) and glucose (1–25 µg assay−1). Figure S5. Analysis of the linearity observed for the standard LOLAC assay as described when the sample volume employed for a range of commercial dairy products was varied from 0.1 to 0.4 mL. [file JSFA-99-947-s001.docx]

|  |
| --- |
| Supporting Information |
|  |
|  |
|  |
|  |

|  |
| --- |

**List of Supplementary Figures**

[Figure S1: Dilution factor calculation for liquid and solid samples based on the recommended procedures in Section 2.5.1 to 2.5.3. 2](#_Toc521504659)

[Figure S2: Absorbance response observed for X µg lactose in the standard LOLAC assay procedure (NADH response) and in the modified LOLAC assay procedure using the glucose detection system described in Section 2.8. 3](#_Toc521504660)

[Figure S3: HPAEC-PAD chromatograph obtained for the analysis of the DP2 fraction obtained from Vivinal GOS as described in Section 2.3. 4](#_Toc521504661)

[Figure S4: Analysis of the linearity observed for the standard LOLAC assay as described in Section 2.5 using lactose (0.2-50 µg/assay) and glucose (1-25 µg/assay). 5](#_Toc521504662)

[Figure S5: Analysis of the linearity observed for the standard LOLAC assay as described in Section 2.5 when the sample volume employed for a range of commercial dairy products was varied from 0.1 mL to 0.4 mL 6](#_Toc521504663)


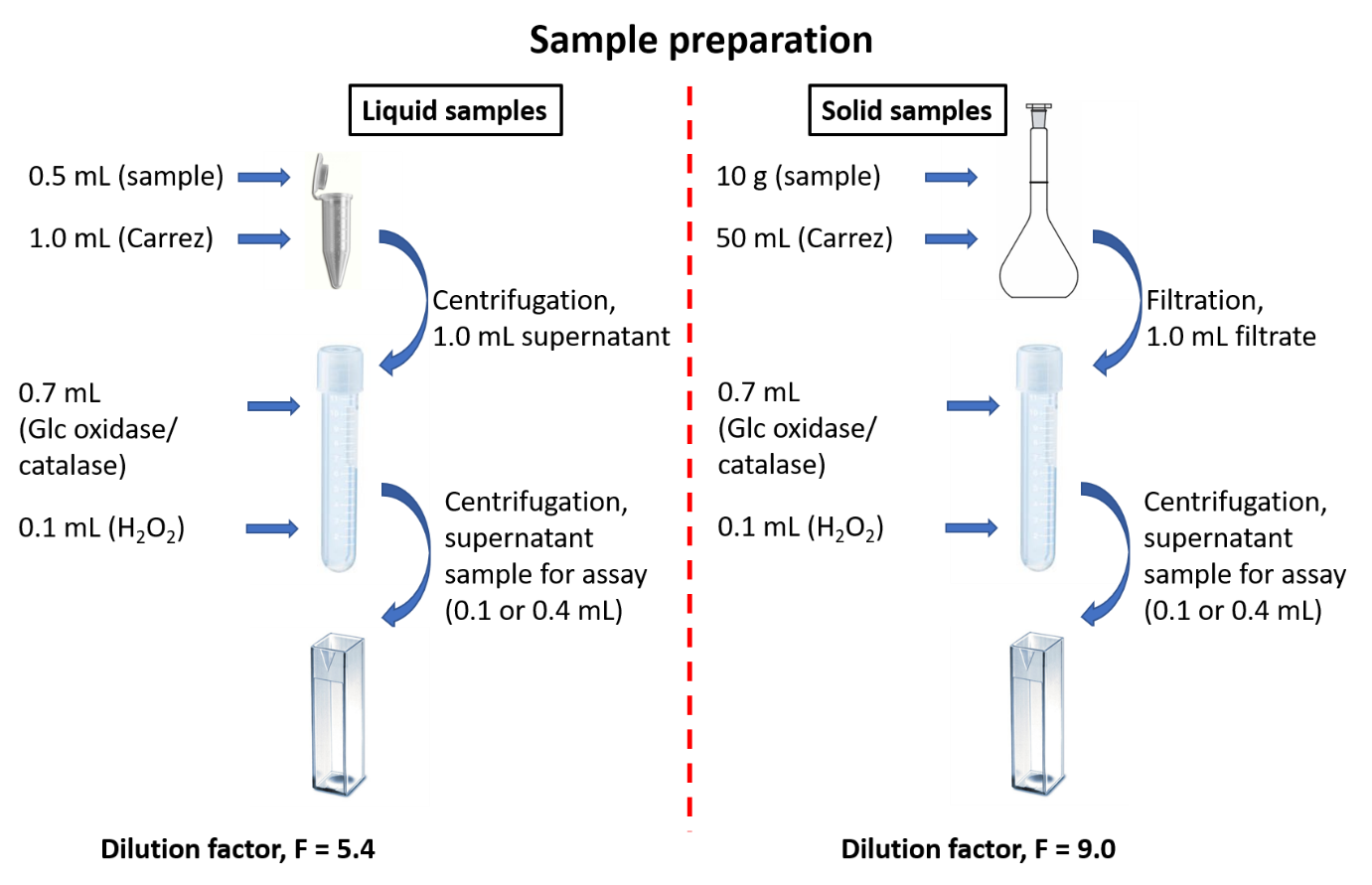


# Figure S1: Dilution factor calculation for liquid and solid samples based on the recommended procedures in Section 2.5.1 to 2.5.3.





# Figure S2: Absorbance response observed for X µg lactose in the standard LOLAC assay procedure (NADH response) and in the modified LOLAC assay procedure using the glucose detection system described in Section 2.8.


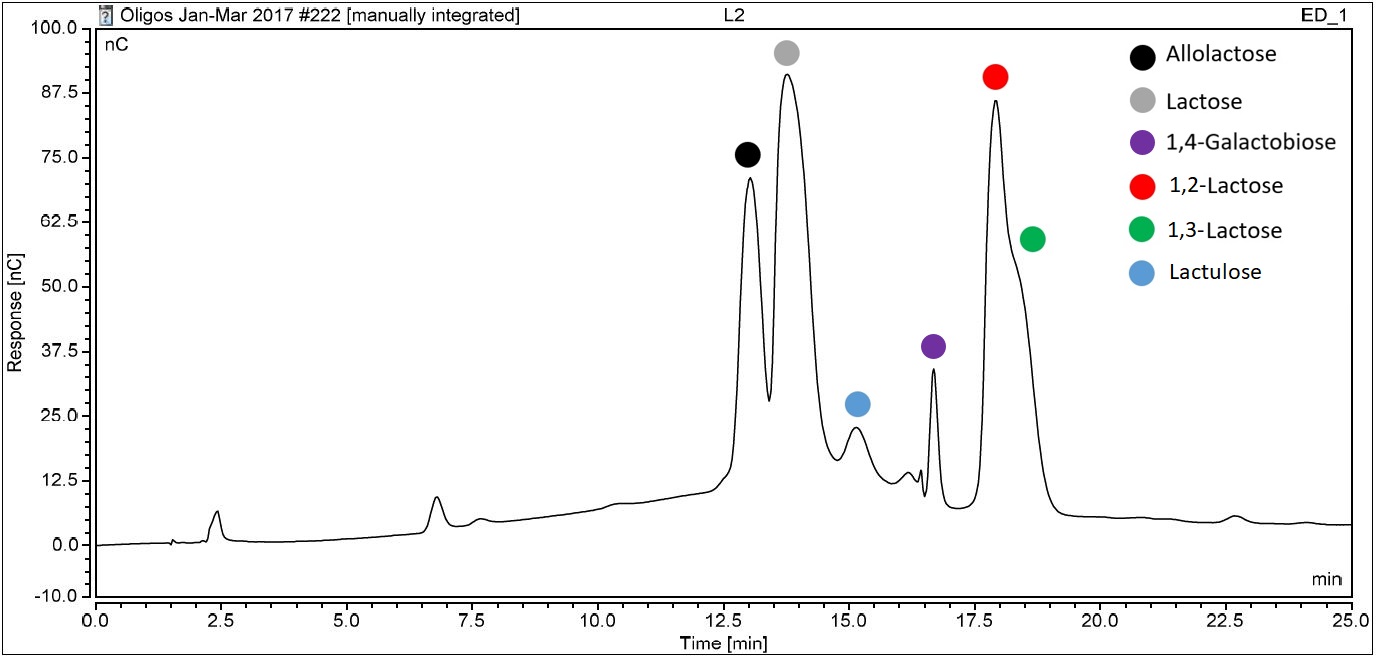


# Figure S3: HPAEC-PAD chromatograph obtained for the analysis of the DP2 fraction obtained from Vivinal GOS as described in Section 2.3.


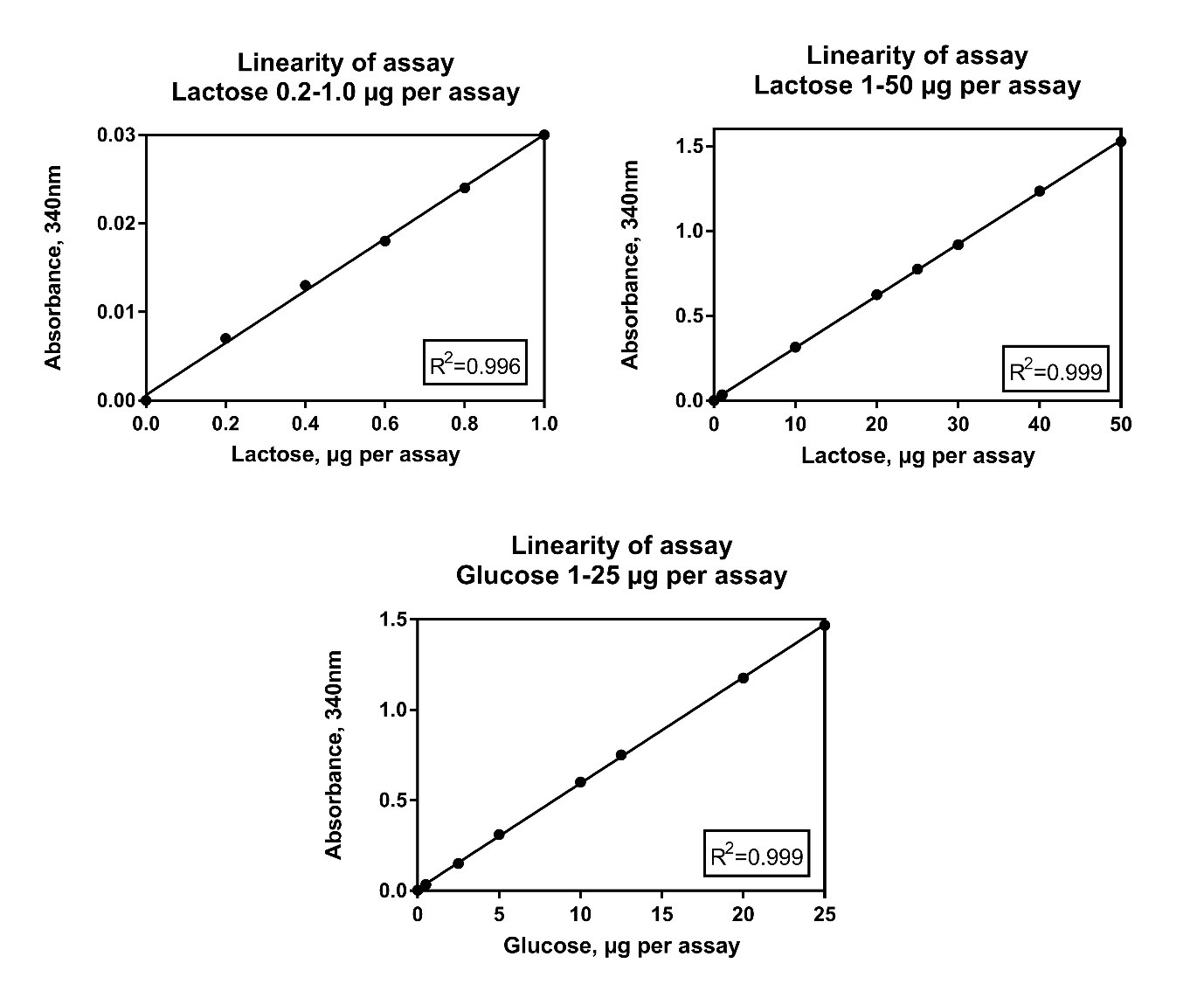


# Figure S4: Analysis of the linearity observed for the standard LOLAC assay as described in Section 2.5 using lactose (0.2-50 µg/assay) and glucose (1-25 µg/assay).


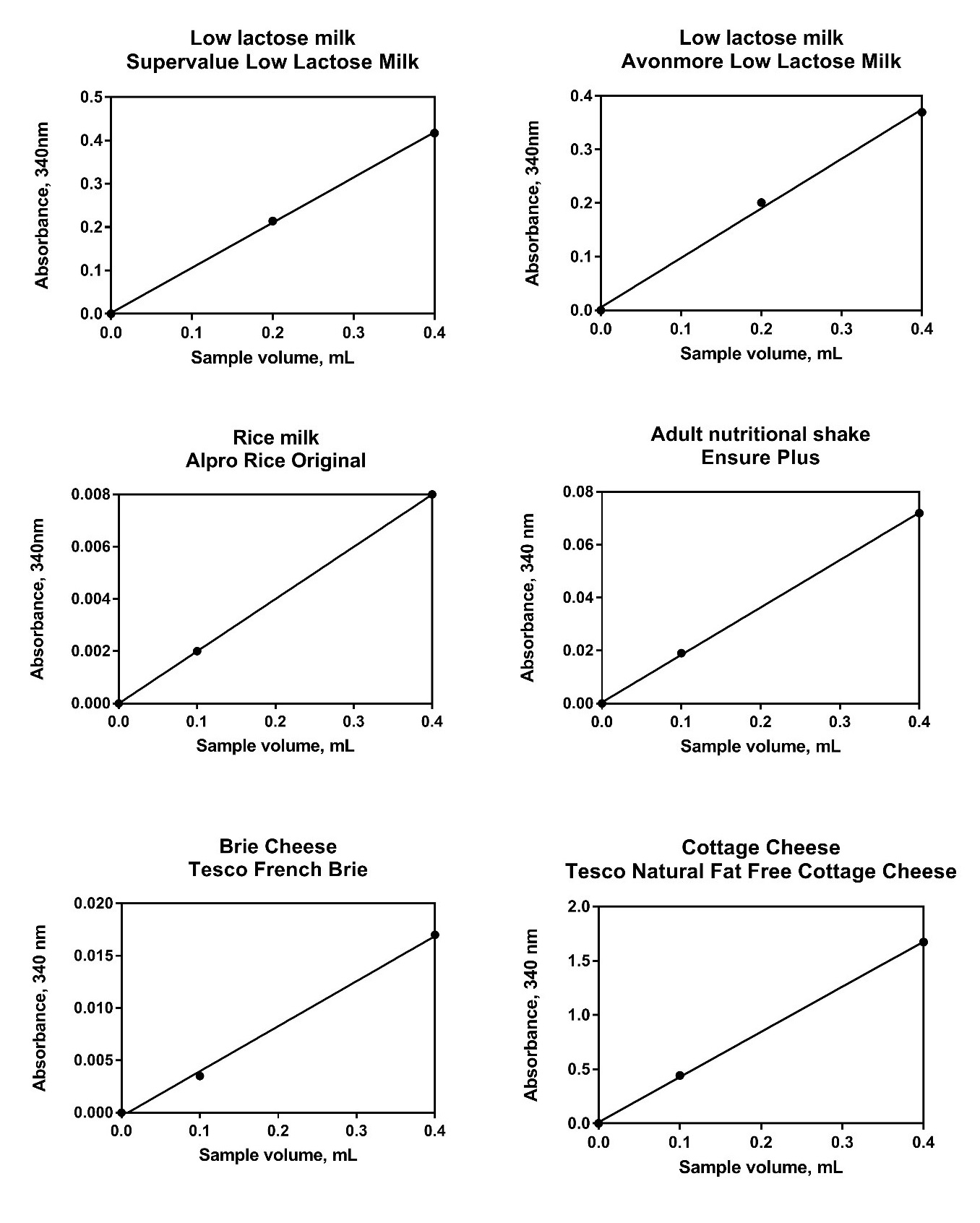


# Figure S5: Analysis of the linearity observed for the standard LOLAC assay as described in Section 2.5 when the sample volume employed for a range of commercial dairy products was varied from 0.1 mL to 0.4 mL
